# Supplementary material for: Reduced Endocannabinoid Tone in Saliva of Chronic Orofacial Pain Patients
Source: Molecules. 2022 Jul 21;27(14):4662. doi: 10.3390/molecules27144662 (PMC9322033; doi:10.3390/molecules27144662)
Supplement: Supplementary file 1 [file molecules-27-04662-s001.zip › molecules-1790838-supplementary.pdf]

Table S1. Pain group: Specific diagnosis, group diagnosis and medications.

Pain group	Drug	Diagnosis
Musculoskeletal	n	MMP
Musculoskeletal	Amitriptyline	TMD
Musculoskeletal	Amitriptyline	MMP
Musculoskeletal	n	TMD
Musculoskeletal	Amitriptyline	MMP
Musculoskeletal	n	MMP
Musculoskeletal	n	MMP
Musculoskeletal	Nortriptyline	MMP
Musculoskeletal	n	MMP
Musculoskeletal	n	TMJ
Musculoskeletal	n	MMP
Musculoskeletal	Duloxetine	TMD
Musculoskeletal	n	TMD
Musculoskeletal	n	TMD
Musculoskeletal	Amitriptyline	MMP
Musculoskeletal	n	MMP
Musculoskeletal	Amirtiptyline	MMP
Musculoskeletal	Nortriptyline	MMP
Musculoskeletal	Amirtiptyline	MMP
Musculoskeletal	Nortriptyline	TMD
Musculoskeletal	n	MMP
Musculoskeletal	n	MIG
Musculoskeletal	Amirtiptyline	MMP
Musculoskeletal	Nortriptyline	MMP
Musculoskeletal	Amirtiptyline	MMP
Musculoskeletal	Clonazepam	MMP
Musculoskeletal	Nortriptyline	MMP
Neuropathic	Carbamazepine	TN
Neuropathic	Pregabalin, Carbamazepine	TN
Neuropathic	Pregabaline	PTN
Neuropathic	Pregabaline	PTN
Neuropathic	Duloxetine	PHN
Neuropathic	Carbamazepine	TN
Neuropathic	Amirtiptyline, Pregabalin	PTN
Neuropathic	n	BMS, PTN
Neuropathic	Carbamazepine	TN
Neuropathic	Amirtiptyline	PTN
Neuropathic	Amirtiptyline	PIFP
Neuropathic	n	PHN
Neuropathic	Duloxetine and more	PTN
Neuropathic	Pregabaline	TN
Neuropathic	Clonazepam	PTN BMS
Neuropathic	n	BMS
Neuropathic	n	BMS
Neuropathic	n	TN
Neuropathic	Pregabaline	PIFP
Neuropathic	Carbamzepine	TN
Neuropathic	Clonazepam	BMS

Neuropathic	Pregabalin	PTN
Neuropathic	Carbamazepine	TN
Neuropathic	n	BMS
Neuropathic	Carbamazepine	TN
Neuropathic	Nortriptyline	PTN
Neuropathic	Clonazepam	BMS
Neuropathic	Pregabaline	PTN
Neuropathic	Carbamazepine	TN
Neurovascular	Nortriptyline	TTH
Neurovascular	Topiramte	MIG
Neurovascular	Topiramate, Amitriptyline	MIG
Neurovascular	n	TTH
Neurovascular	n	TTH
Neurovascular	Nortriptyline	MIG
Neurovascular	Topiramte	NVOP
Neurovascular	tripatans abbortive	MIG
Neurovascular	topamax	MIG
Neurovascular	Nortriptyline	TTH
Neurovascular	Topiramat	NVOP
Neurovascular	Amirtiptyline	NVOP
Neurovascular	Nortriptyline	TTH
Neurovascular	topamax 100 mg+rizalit	NVOP
Neurovascular	Valproic acid	MIG
Neurovascular	Triptans abbortive	MIG
Neurovascular	Amirtiptyline	NVOP
Neurovascular	Amirtiptyline	NVOP
Neurovascular	Amirtiptyline, Pregabalin	MIG
Neurovascular	Pregabaline	MIG
Neurovascular	Topiramat	MIG
Neurovascular	multiple drugs	MIG
Neurovascular	n	MIG
Neurovascular	Amitriptyline, Topitrimate	TTH MIG
Neurovascular	Topiramte	MIG PTN
Neurovascular	trigger point Injection	MIG
Neurovascular	n	NVOP
Neurovascular	Topiramte	NVOP
Neurovascular	Nn	MIG, MMP
Muscolesceletal	Nortriptyline	A typical MMP

n - none
